# Characterization of *Neopestalotiopsis* Species Associated with Mango Grey Leaf Spot Disease in Sinaloa, Mexico

**DOI:** 10.3390/pathogens9100788

**Published:** 2020-09-25

**Authors:** Saida S. Gerardo-Lugo, Juan M. Tovar-Pedraza, Sajeewa S. N. Maharachchikumbura, Miguel A. Apodaca-Sánchez, Kamila C. Correia, Carlos P. Sauceda-Acosta, Moisés Camacho-Tapia, Kevin D. Hyde, Najat Marraiki, Abdallah M. Elgorban, Hugo Beltrán-Peña

**Affiliations:** 1 Facultad de Agronomía, Universidad Autónoma de Sinaloa, Culiacán, Sinaloa 80090, Mexico; saida.gl@mochis.tecnm.mx; 2 Laboratorio de Fitopatología, Coordinación Culiacán, Centro de Investigación en Alimentación y Desarrollo, Culiacán, Sinaloa 80110, Mexico; juan.tovar@ciad.mx; 3 School of Life Science and Technology, University of Electronic Science and Technology of China, Chengdu 611731, China; 4 Departamento de Ciencias Naturales y Exactas, Unidad Regional Los Mochis, Universidad Autónoma de Occidente, Los Mochis, Sinaloa 81223, Mexico; apodacasma@yahoo.com; 5 Centro de Ciências Agrárias e da Biodiversidade, Universidade Federal do Cariri, Crato, Ceará 63.133-610, Brazil; Kamila.correia@ufca.edu.br; 6 Facultad de Agricultura del Valle del Fuerte, Universidad Autónoma de Sinaloa, Ahome, Sinaloa 81110, Mexico; saucedap@uas.edu.mx; 7 Laboratorio Nacional de Investigación y Servicio Agroalimentario y Forestal, Universidad Autónoma Chapingo, Texcoco 56230, Estado de México, Mexico; camacho.moises@colpos.mx; 8 Center of Excellence in Fungal Research, Mae Fah Luang University, Chiang Rai 57100, Thailand; kdhyde3@gmail.com; 9 Department of Botany and Microbiology, College of Science, King Saud University, P.O. Box 2455, Riyadh 11451, Saudi Arabia; najat@ksu.edu.sa (N.M.); aelgorban@ksu.edu.sa (A.M.E.)

**Keywords:** *Mangifera indica*, morphology, pathogenicity, virulence, phylogeny

## Abstract

Mango is one of the most popular and nutritious fruits in the world and Mexico is the world’s largest exporter. There are many diseases that directly affect fruit yield and quality. During the period 2016–2017, leaves with grey leaf spots were collected from 28 commercial mango orchards distributed in two main production areas in Sinaloa State of Mexico, and 50 *Neopestalotiopsis* isolates were obtained. Fungal identification of 20 representative isolates was performed using morphological characterization and phylogenetic analysis based on the internal transcribed spacer (ITS) region of ribosomal DNA, part of the translation elongation factor 1-alpha (TEF) and the β-tubulin (TUB) genes. Phylogenetic analysis indicated that the 20 isolates from this study formed four consistent groups, however, overall tree topologies do not consistently provide a stable and sufficient resolution. Therefore, even though morphological and phylogenetic separation is evident, these isolates were not assigned to any new taxa and were tentatively placed into four clades (clades A–D). Pathogenicity tests on detached mango leaves of cv. Kent showed that the 20 isolates that belong to the four *Neopestalotiopsis* clades from this study and induce lesions on mango leaves. This is the first report of species of *Neopestalotiopsis* causing mango grey leaf spot disease in Mexico.

## 1. Introduction

Mango (*Mangifera indica* L.; Anacardiaceae) is the fifth most economically important tropical fruit in the world [1,2]. The mango fruit is appreciated for its unique flavor and nutritional value and is consumed in juices, beverages, jams and as a fresh fruit [3]. Over 90 countries cultivate mango, [4] Asia is the leading producer with 75% of the world’s production [5], but Mexico is the world’s largest exporter [6]. In Mexico, mango is the second tropical fruit of economic importance and during 2019, the production volume was 2,087,359 t in 193,549 ha, cultivated in 23 states, with Sinaloa as the largest producing state. The main cultivars produced in Mexico are Ataulfo, Kent, Manila, Tommy Atkins, Keitt, and Haden [7].

Diseases caused by fungi are the main limiting factor for mango production worldwide [8]. In Mexico, powdery mildew (*Pseudoidium anacardii*), anthracnose (*Colletotrichum* spp.), mango malformation disease (*Fusarium* spp.), stem-end rot (*Lasiodiplodia* spp. and *Neofusicoccum* spp.) and grey leaf spot (*Pestalotiopsis mangiferae*) are the most important diseases [9,10,11,12,13]. The grey leaf spot of mango has been reported in Australasia, Europe, USA and is widespread in Africa and Asia [12,14,15,16]. Initially, irregular yellow-to-brown spots appear on leaves and then turn white to grey and coalesce to form patches with abundant black acervuli [15,16]. In Mexico, grey leaf spot disease was observed in 0.3 to 2.0% of fruit cv. Manila [12].

The genus *Neopestalotiopsis* was segregated from *Pestalotiopsis* based on the conidial morphology and the phylogenetic analysis of the nuclear ribosomal RNA gene [17]. Generally, the colour of the median conidial cells enables the differentiation of the three genera [17]. The conidia with versicolored median cells belong to the genus *Neopestalotiopsis*, which seems to have evolved from the lineage of *Pseudopestalotiopsis* whose members have dark concolorous conidial median cells, while *Pestalotiopsis* presents the three light concolorous conidial median cells [17]. The morphology of the pestalotiopsis-like taxa varies depending on the environment and the host in which they were isolated. Therefore, the separation of species through phenotypic characteristics is difficult [18].

Pestalotiopsis-like taxa are important plant pathogens in tropical and subtropical regions, infecting diverse crops and causing leaf spots, dry flowers, root rot, trunk diseases, fruit rot and fruit scab [18,19,20,21,22,23,24,25,26,27]. Several pestalotiopsis-like species are responsible for the grey leaf spot of mango, including *Neopestalotiopsis clavispora*, *N. egyptiaca*, *Pestalotiopsis anacardiacearum*, *P. mangiferae* and *P. uvicola* [15,16,28,29,30]. Previously, Noriega-Cantú et al. [12] reported *P. mangiferae* as the causal agent of grey leaf spot in mango in Mexico, and the three median cells of the conidia of this species were observed as dark brown, which was not comparable with the characteristics of the genus *Pestalotiopsis.* However, the multi-locus sequence data proposed by Maharachchikumbura et al. [31] were not applied in their work, and the species was not confirmed to belong to *Neopestalotiopsis*.

During the period 2016–2017, symptoms of grey leaf spots were observed in commercial mango orchards distributed in the Sinaloa state of Mexico with high incidence and disease intensity up 8%. Therefore, the aims of this study were to (1) identify fungal taxa associated with grey leaf spot of mango in Sinaloa by a combination of morphological characterization and phylogenetic analyses and (2) determine their pathogenicity and virulence on detached mango leaves.

## 2. Results

### 2.1. Fungal Isolates

**Figure 1:**
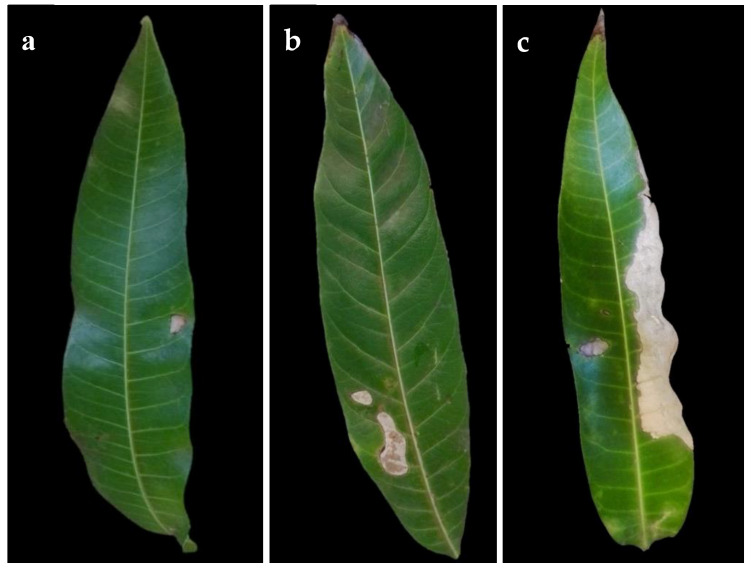
Symptoms of grey spot disease on naturally infected mango leaves. (**a**) Small lesion (early symptom) (**b**) Lesions coalescing. (**c**) Leaf blight (advanced symptom).

Isolation from symptomatic mango leaves with grey blight resulted in numerous fungal pestalotiopsis-like isolates being the most common (Table 1). Other fungi isolated from symptomatic leaves were *Alternaria* spp. and *Colletotrichum* spp. Based on the initial phenotypic characterization following the morphological traits described by Maharachchikumbura et al. [17], these isolates were found to belong to the genus *Neopestalotiopsis.* Since our effort was on pestalotiopsis-like species, we excluded the remaining isolates from further analysis. A total of 50 *Neopestalotiopsis* isolates from symptomatic mango leaves were obtained. Initially observed symptoms were irregular brown lesions not delimited by the veins (Figure 1a). The spots enlarged rapidly and form grey lesions or patches in older lesions, in some cases, with dark acervuli (Figure 1b,c). No symptoms were observed on fruits.

### 2.2. Morphological and Cultural Characteristics

**Figure 2:**
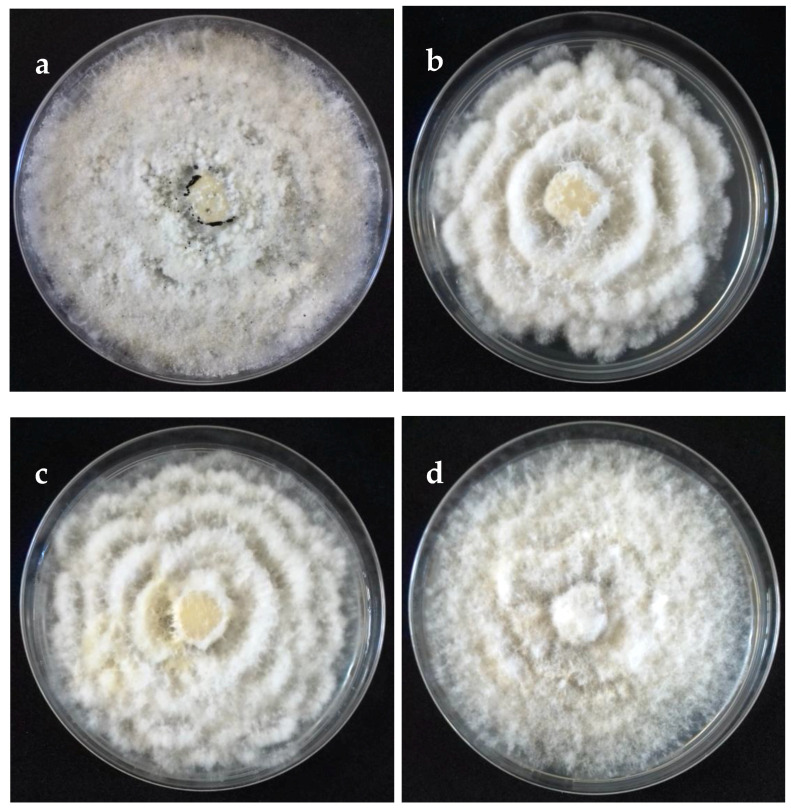
Colonies on potato dextrose agar (PDA) (front) of selected isolates representative of *Neopestalotiopsis* clades at 10 days after incubation. (**a**) Clade A (isolate FAVF 160). (**b**) Clade B (isolate FAVF 157). (**c**) Clade C (isolate FAVF 162). (**d**) Clade D (isolate FAVF 169).

**Figure 3:**
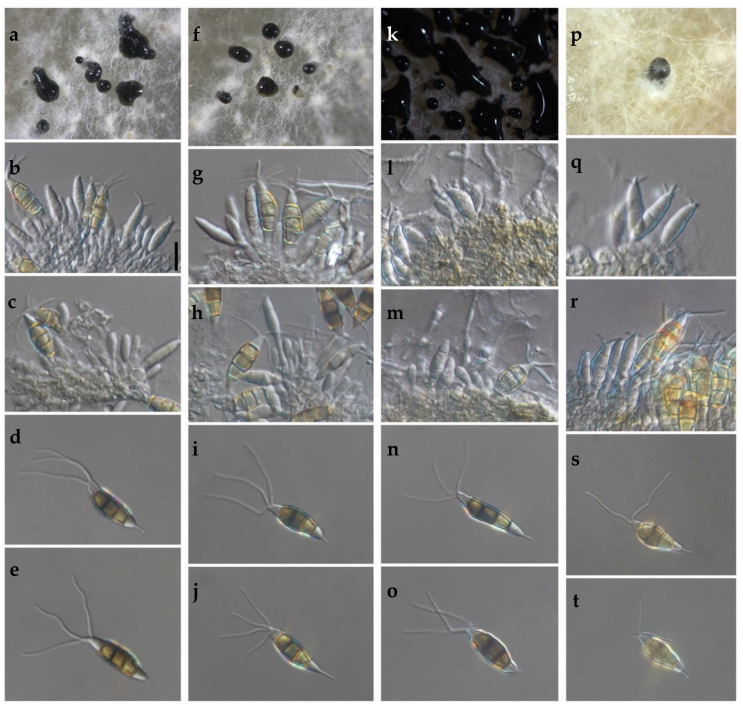
(**a**–**t**) Morphological features of selected isolates of *Neopestalotiopsis* Clades. (**a**–**e**) Clade A (isolate FAVF 198). (**f**–**j**) Clade B (isolate FAVF 204). (**k**–**o**) Clade C (isolate FAVF 162). (**p**–**t**) Clade D (isolate FAVF 167). (**a**,**f**,**k**,**p**) Conidiomata on PDA. (**b**,**c**,**g**,**h**,**l**,**m**,**q**) Conidiogenous cells. (**d**,**e**,**i**,**j**,**n**,**o**,**r**–**t**) Conidia. Scale bars (**b**) = 20 μm. Scale bar of (**b**) applies to (**c**–**e**,**g**–**j**,**l**–**o**,**q**–**t**).

The cultural characteristics of colonies in medium potato dextrose agar (PDA) (Becton Dickinson, Mexico) showed variation between different clades and isolates of the same clade (Table 2, Figure 2). Colonies were classified into four morphotypes. The first morphotype was present in all four clades. The second morphotype was exhibited by the Clade A and Clade B. The third morphotype was only observed in Clade C. The fourth morphotype was identified in Clade B and Clade D. This last clade was the most variable concerning the type of colony. Acervuli were observed in Clade A, Clade C, and Clade D on the PDA medium (Figure 2). The mycelial growth rate presented significant differences (*p* ≤ 0.05) between clades, Clade A was different from Clade B, but similar to Clade C and Clade D (Table 2). Conidia of the four clades were fusoid, 4-septate, hyaline end cells, versicolour median cells (two upper medium cells fuliginous and lower median cell pale brown) (Figure 3). The conidia presented 2–4 tubular apical appendages, unbranched, attached at the top of the basal cell, and one cylindrical basal appendage (Figure 3). In the length of the conidium, Clade D was different (*p* ≤ 0.05) to the rest of the clade, showing the largest conidia. The widest conidia were formed in the Clade B and Clade C with significant differences (*p* ≤ 0.05) when compared with the Clade A and Clade D (Table 2). The length of apical appendages was statistically different (H = 37.24, *p* ≤ 0.05) between clades, the longest apical appendages were observed in the Clade C. The Clade B presented the longest basal appendages and was significantly different (H = 81.89, *p* ≤ 0.05) to the other clades. The most common number of apical appendages was three with no significant difference between clades (Table 2).

### 2.3. Phylogeny

**Figure 4:**
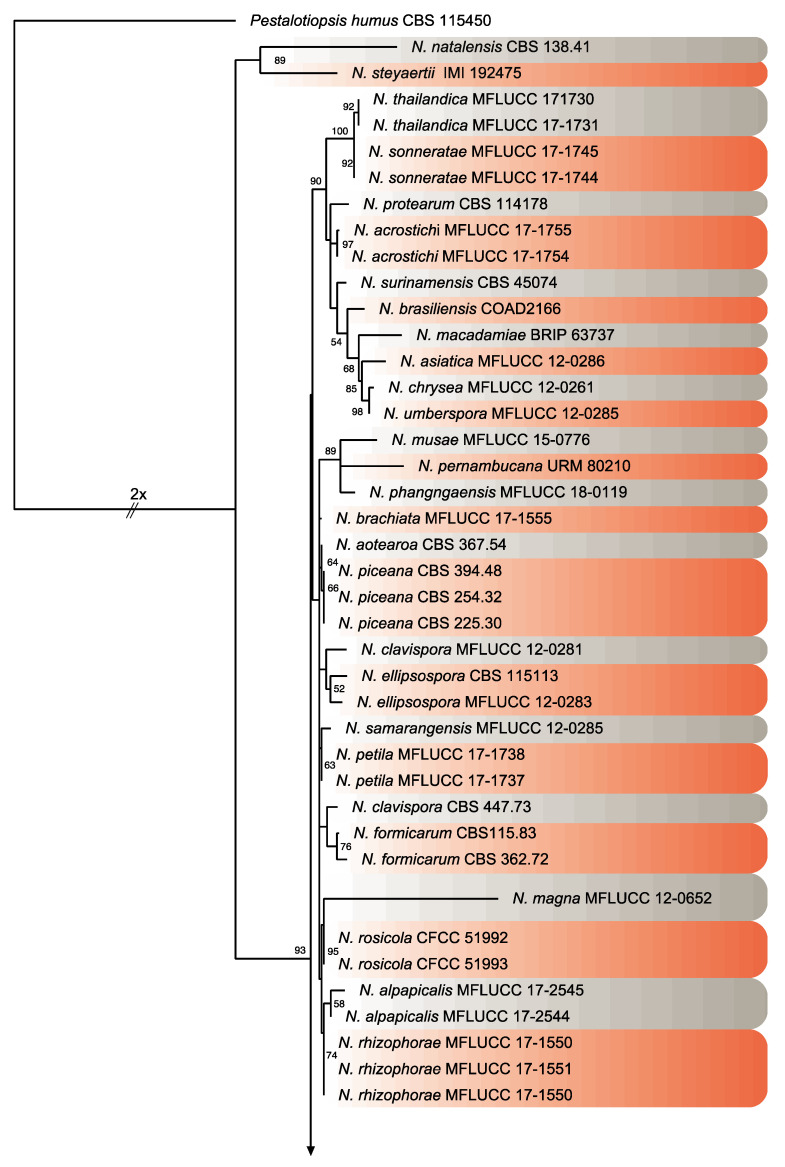

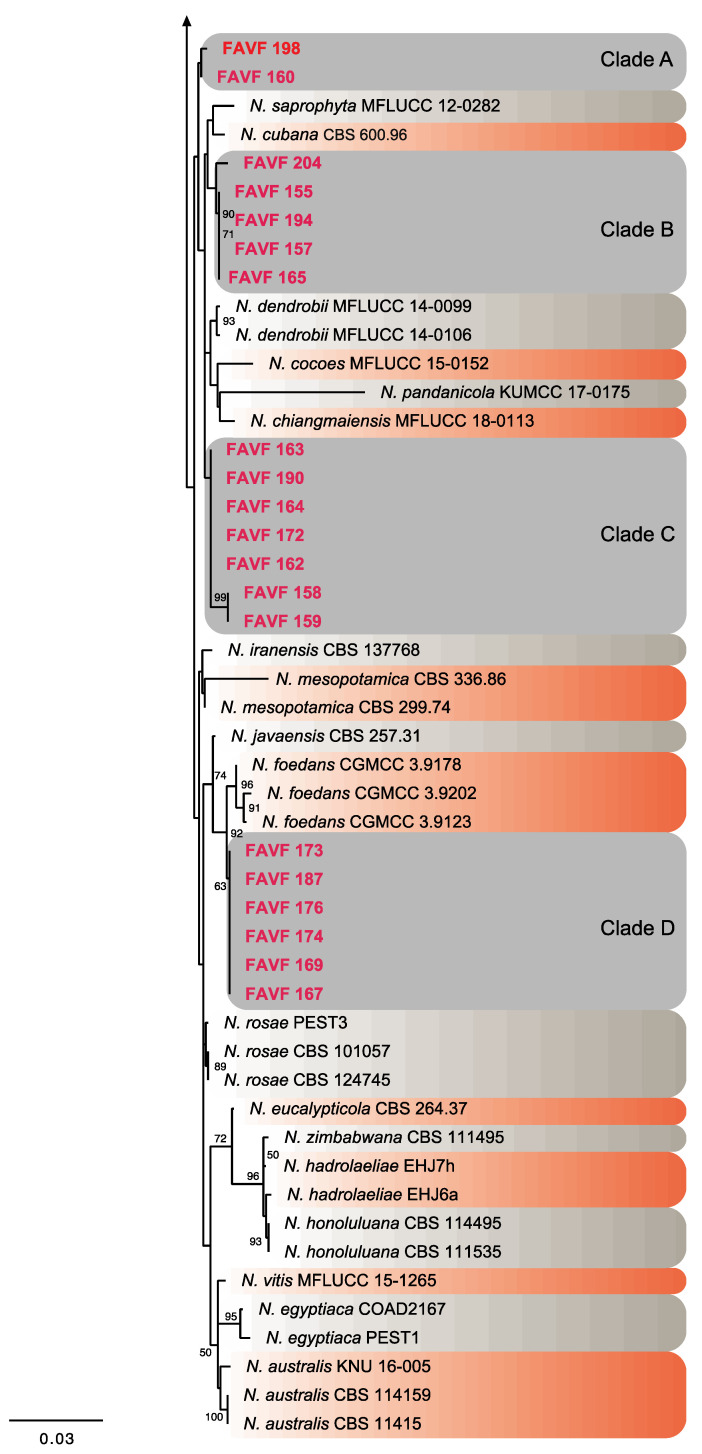
Consensus phylogram tree resulting from a Maximum Likelihood (ML) analysis of the combined internal transcribed spacer (ITS), translation elongation factor 1-alpha (TEF) and β-tubulin (TUB) sequence alignments of *Neopestalotiopsis* isolates. Bootstrap support values above 50% are indicated at the nodes. The sequences derived from the present study are written in red. The scale bar represents the expected number of changes per site. The tree was rooted to *Pestalotiopsis humus* (CBS 115450).

**Figure 5:**
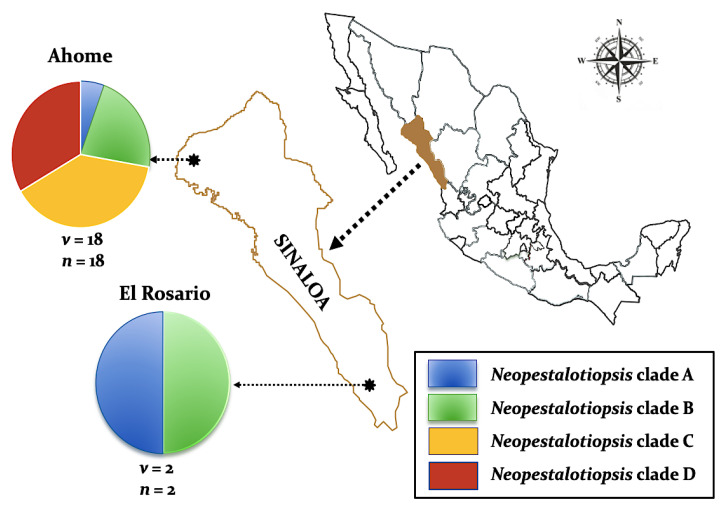
Distribution of four *Neopestalotiopsis* clades associated with mango grey leaf spot in 20 orchards distributed in the municipalities of Ahome and El Rosario, Sinaloa, Mexico. Circles represent association frequency of each clade with each population sampled, “*v*” is the number of commercial orchards sampled in each population and “*n*” is the number of isolates analyzed in each population.

Twenty isolates of *Neopestalotiopsis* were selected as representative for phylogenetic analysis (Table 1). The combined internal transcribed spacer (ITS) + the β-tubulin (TUB) + the translation elongation factor 1-alpha (TEF) alignment was used to resolve the species relationship in the genus *Neopestalotiopsis* (Figure 4). The combined datasets consisted of 91 taxa, including *Pestalotiopsis humus* (CBS 115450) as the outgroup taxon. The alignment contained 1500 characters (521 for ITS, 483 for TUB, and 496 for TEF) and the combined datasets resulted in a best scoring RAxML tree with a final maximum likelihood (ML) optimization value of likelihood of −6532.281881, shown in Figure 4. The overall topology of our tree is comparable to that of earlier studies [17,20,32]. However, the overall branch-length support values were lower in most clades and recent studies have shown that the use of combined ITS, TEF, and TUB sequences to resolve numbers of cryptic species in pestalotiopsis-like taxa have some shortcomings. Therefore, even though the isolates from this study formed several consistent clades in the ML analysis, they were organized into four tentative clades (Clades A–D) and were not assigned to any taxa because the sequence data were insufficient to determine species boundaries in these isolates. The Clade D was the most frequently recorded in this study with seven isolates, followed by Clade C, B, and A. However, isolates belonging to Clade A and B were present in both municipalities of Sinaloa (Mexico), while isolates from Clade C and D were only recorded in the municipality of Ahome (Figure 5).

### 2.4. Pathogenicity and Virulence on Mango Leaves

**Figure 6:**
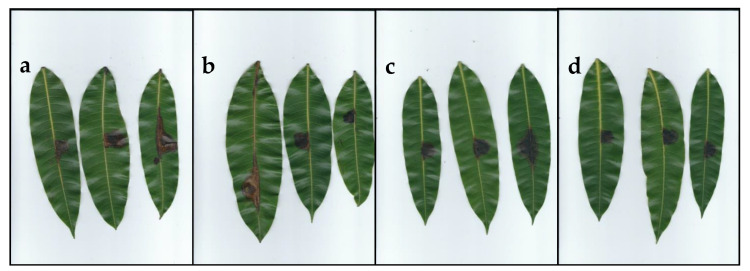
Pathogenicity test of *Neopestalotiopsis* isolates on mango leaves after 10 days of inoculation. Leaves inoculated with: (**a**) Clade A (isolate FAVF 198). (**b**) Clade B (isolate FAVF 204). (**c**) Clade C (isolate FAVF 162). (**d**) Clade D (isolate FAVF 187).

All 20 tested isolates of *Neopestalotiopsis* distributed in the four clades were pathogenic on detached mango leaves cv. Kent. Inoculated leaves developed irregular brown lesions (Figure 6) with abundant black acervuli, 10 days after inoculation (DAI), and the lesions turned greyish 17 DAI, similar symptoms to those observed in naturally infected mango leaves. On the other hand, control leaves remained symptomless. Fungal colonies were re-isolated from all symptomatic leaves and were found to be morphologically identical to the original isolates inoculated, thus fulfilling Koch’s postulates. These results confirmed that *Neopestalotiopsis* is the causal agent of grey leaf spot in the production areas from Sinaloa. The average severity on artificially inoculated mango leaves was 4.16, 3.01, 4.25 and 3.24% of the total leaf area for *Neopestalotiopsis* Clade A, Clade B, Clade C and Clade D, respectively, without difference between clades (*p* ≥ 0.05). Additionally, the virulence of the 20 *Neopestalotiopsis* isolates did not show significant differences (*p* ≥ 0.05) and the growth rate was not correlated with the lesion area (*r* = 0.13, *p* ≥ 0.05).

## 3. Discussion

Pestalotiopsis-like fungi are difficult to differentiate based on morphological characters, because they vary according to the nature of the isolation and environmental conditions [17]. Based on the conidial characteristics, such as 4-septate, fusiform conidia, and versicolored median cells, it was possible to distinguish the 20 isolates into the genus *Neopestalotiopsis* and separate them from other pestalotiopsis-like genera [17,18]. The combination of host, symptoms, morphological characters and multi-locus phylogeny provides the tools for the species-level recognition of *Neopestalotiopsis*; however, using analysis of the combined ITS, TUB and TEF sequence data did not allow differentiation at the species level. The overall topology of our phylogenetic tree was not sufficiently supported, similar to that published in previous studies that maintained the *Neopestalotiopsis* isolates as *Neopestalotiopsis* spp. [17,32,33,34,35]. Although multi-locus phylogeny is important in species differentiation, there is still a lack of species deposited in public culture collections and only a fraction of DNA has been sequenced [36].

The pathogenicity test confirmed that all inoculated isolates were pathogenic on mango leaves cv. Kent, inducing brown and grey lesions with conidia exuding a cirrus from black acervuli. The symptoms were similar to those reported on mango grey leaf spot disease caused by pestalotiopsis-like fungi in Italy and China [15,16,29,37]. No symptoms were observed on fruits in mango orchards from Sinaloa. Perhaps this is due to the application of fungicides such as carbendazim, mancozeb, and copper oxychloride for the control of anthracnose in the mango producing areas of Sinaloa, which begins in pre-flowering until a few days before it harvests. These fungicides have been effective in controlling foliar diseases caused by *Pestalotiopsis mangiferae,* and *Pestalotia anacardii* in mango and *Neopestalotiopsis cubana* in rubber tree (*Hevea brasilensis*) [12,38,39]. It is necessary to carry out further studies to determine the susceptibility of the mango cultivars as well as to evaluate fungicides for controlling grey leaf spot disease in Mexico.

In this study, the incidence (% orchards infected) of mango grey leaf spot disease was high, with 100% in both municipalities of Sinaloa. The incidence at the field level (% tree infected) was not evaluated, but 70% to 100% have been reported in Italy [16]. Similar results have been reported for grey leaf spot disease with premature defoliation by pestalotiopsis-like fungi in other hosts, like *Jatropha curcas*, *Canthium dicoccum*, *Vigna unguiculata*, *Cocos nucifera* and *Vacciniea corymbosum* [40,41,42,43,44]. However, premature defoliation associated with grey leaf spot disease was not observed in the present study. Although some mango diseases such as anthracnose, powdery mildew and mango malformation [10,12,13] may have a higher incidence and severity in the orchards of Sinaloa, attention should be paid to emerging diseases such as grey spot.

*Neopestalotiopsis* Clades A–D showed no significant differences in disease severity under controlled conditions (humidity and temperature), but this could be different under field conditions, as reported in mango orchards from Taiwan and mainland China infected with grey spot disease [15,16]. There were no significant differences in virulence between clades A–D, nor between virulence and geographic origin of the isolates as observed in China with mango affected by pestalotiopsis-like species [37]. The growth rates of the isolates in this study are similar to the grey leaf spot disease reported by pestalotiopsis-like species [15,37]. Although the growth rates were almost the same in all the clades, only a field study could describe the behavior, estimate the rate of disease and determine the disease progression as a percentage of damage [45]. The symptoms of fungal diseases developed in mango can vary greatly, according to various factors, such as cultivar and environmental conditions [46]. Therefore, further research is needed to determine the different environmental conditions that affect the development of symptoms. Knowledge about the variation of virulence, its spatial distribution, and the development of the disease will provide the tools to establish integrated disease management.

This study represents the first detailed investigation of morphology, phylogeny and pathogenicity of *Neopestalotiopsis* species causing mango leaf spot in Mexico. The variation of the specific isolates in each of the clades described in this work, provides information on a threat to the mango industry, either due to its development of resistance to fungicides, as well as possible variations in virulence that it could have in the different cultivars and climatic conditions. Therefore, it is crucial to continue studying these clades in order to have knowledge of their epidemiology and their impact on mango productivity. To our knowledge, this is the first report of genus *Neopestalotiopsis* causing grey leaf spot of mango in Mexico.

## 4. Materials and Methods

### 4.1. Sample Collection

During the surveys carried out in 2016 and 2017, mango leaves exhibiting typical symptoms of grey spots (Figure 1) were collected from different cultivars in 28 commercial mango orchards distributed in the municipalities of Ahome in the north and El Rosario in the south, the two main production areas in Sinaloa, Mexico. The GPS readings were taken, and the collections were brought to the laboratory in Ziplock plastic bags.

### 4.2. Isolation and Purification

Symptomatic leaves were washed with neutral soap and surface sterilized with 90% ethanol for 30 s. The pieces of leaves (4 mm^2^) from the margins between the necrotic and healthy tissues were surface sterilized with 1% sodium hypochlorite for 60 s. The leaf pieces were washed with sterile distilled water and excess liquid was removed by sterile filter paper. The leaf pieces were placed in PDA plates and incubated at 25 °C for 3 days in darkness. Mycelial plugs from the edge of fungal hyphae developing from the tissues were aseptically transferred to fresh PDA and were incubated at 25 °C for 7 days in darkness. Monoconidial cultures were obtained using the method described by Zhang et al. [47]. In briefly fruiting bodies were crushed to separate the spores and obtain a spore suspension. The suspension was inoculated on to fresh PDA plates and the germinating conidia were aseptically transferred to fresh PDA plates. The purified cultures were stored in tubes with PDA covered with sterile mineral oil at 20 °C.

### 4.3. Morphological Characterization and Growth Rate

Macroscopic and microscopic characteristics were examined for 20 representative isolates of *Neopestalotiopsis* from mango collection sites in Sinaloa. To determine the growth rate of each isolate, mycelial plugs (12 mm diam.) were taken from 3-days-old cultures and placed on PDA. The plates were incubated at 25 °C in darkness. Colony diameters of three biological replicates of each isolate were daily examined over 5 days. The growth rate (*k_r_*) was calculated with the linear growth function *y* = *k_r_ x* + *c* (where *y* is the distance, *x* is the time and *c* is the constant factor) and was expressed in mm day^−1^ [48]. After 10 days, colony growth characteristics, including colour of the colony and conidial masses were recorded. The experiment was conducted twice. Macro-morphological characters of the conidiomata were photographed by a Carl Zeiss Stemi 508 stereo microscope and conidial characters (*n* = 50) were visualized with a Nikon Eclipse Ni-U, microscope. All images were prepared using Adobe Photoshop CC 2018 and all measurements were made with NIS-element D software. Cultures of each isolate were deposited in the Culture Collection of Phytopathogenic Fungi of the Faculty of Agriculture from El Fuerte Valley (FAVF) at the Autonomous University of Sinaloa (Juan Jose Rios, Sinaloa, Mexico). Statistical analysis was performed with InfoStat^®^ 2017 [49] by ANOVA and the mean values for length and width of conidia were compared using Tukey’s test, while the length of basal and apical appendages was compared using nonparametric Kruskal–Wallis test, in both cases with at a significance level of *p* ≤ 0.05. The correlation between growth rate and lesion area was analyzed by Pearson correlation.

### 4.4. DNA Extraction, PCR Amplification and Sequencing

Total genomic DNA was extracted from fresh fungal mycelia, scraped from the margin of a colony grown on a PDA plate, incubated at 25 °C with a DNeasy Plant Mini Kit (Qiagen, Valencia, CA, USA) following the manufacturer’s protocols. DNA concentrations were quantified using a Nano Drop One (Thermo Fisher Scientific, Madison, WI, USA) and the samples were diluted in sterile water to 100 ng μL^−1^ and stored at −20 °C. The ITS region of ribosomal DNA, part of TEF and TUB genes were amplified using the following pairs of primers: ITS4/ITS5 [50], EF728F/EF1-1567R [51] and BT2A/BT2B [52,53]. PCR reactions for three replicates of each sample were performed with the 25 μL reaction system consisting of 0.4 ng of genomic DNA, 0.04 U of Taq polymerase, 1× buffer, 0.8 μM of each primer, 0.2 mM of dNTPs, and 2.5 mM MgCl_2_. Amplifications were carried out in a Bio-Rad CFX96 thermocycler (Bio-Rad Laboratories, Hercules, CA, USA) with the following profile: an initial denaturation step at 95 °C for 3 min, followed by 35 cycles of denaturation at 95 °C for 30 s, annealing at 55 °C for ITS, 54 °C for the TEF, and 58 °C for TUB for 30 s, extension at 72 °C for 60 s and a final extension at 72 °C for 10 min. The PCR products were visualized on 1% agarose gel electrophoresis stained with ethidium bromide. The amplified PCR products were purified using QIAquick PCR Purification Kit (Qiagen, Valencia, CA, USA) and sequencing was carried out by the Macrogen (Macrogen Inc., Seoul, Korea).

### 4.5. Phylogenetic Analyses

DNASTAR Lasergene SeqMan Pro v.8.1.3 was used to obtain consensus sequences from sequences generated from forward and reverse primers and these were subsequently deposited in the GenBank database (Table 1). Multiple sequence alignments were generated with MEGA v.7.0.26 [54]. An ML analysis was performed using RAxML GUI v. 1.3 [55]. The optimal ML tree search was conducted with 1000 separate runs, using the default algorithm of the program from a random starting tree for each run. The final tree was selected among suboptimal trees from each run by paralleling likelihood scores under the GTR+GAMMA substitution model. The resulting phylograms were illustrated using FigTree v. 1.4.0 (http://tree.bio.ed.ac.uk/software/figtree/).

### 4.6. Pathogenicity and Virulence

Pathogenicity test was carried out on 20 isolates selected based on an initial phylogenetic analysis which resulted in four clades. The pathogenicity of all isolates was tested on mango (cv. Kent) leaves collected from commercial fields at Ahome, Sinaloa. Detached healthy leaves were washed with neutral soap and rinsed with running tap water to remove dirt and were superficially disinfected with 70% ethanol on both sides [27]. Disinfected leaves were wounded in a single site with a sterile toothpick. A mycelial plug (7 mm in diam.) was removed from the margin of 5-days-old growing colonies on PDA at 27 °C and was placed on the wounded site of each leaf. Each *Neopestalotiopsis* isolate was inoculated on three leaves (biological replicates) and the experiment was repeated twice. Mango leaves inoculated with PDA plug without fungal mycelium were used as the control. Inoculated leaves were incubated at 25 °C under a 12/12 h light/darkness on plastic trays lined with two layers of paper towel moistened with sterile distilled water and enclosed in a plastic bag to maintain moisture. Ten DAI, the presence or absence of symptoms on the inoculated leaves served to determine the pathogenicity of the isolates, and the virulence of each isolate was evaluated by determining disease severity. Inoculated leaves were pressed in a botanical press for 15 min and after, digitalized in a scanner (HP Officejet Pro 6830) at 300 dpi resolution. Disease severity was evaluated using digital images and the affected area was calculated by measuring the lesion area (mm^2^) concerning the size of the leaf by digital image analysis, with ImageJ Software (version 1.48r; NIH, Bethesda, MD, USA) [56]. The severity data were analyzed by nonparametric ANOVA with Kruskal–Wallis test using InfoStat^®^ 2017 [49].

## Figures and Tables

**Table 1:** Sequences deposited at the NCBI database and their GenBank accession numbers, from the selected strains isolated in this study.

Taxon	Culture Code	Cultivar	Location	GeneBank Accession Number		
				ITS	TUB	TEF
*Neopestalotiopsis* sp. Clade A	FAVF 198	Tommy Atkins	El Rosario	MT774584	MT782088	MT782108
*Neopestalotiopsis* sp. Clade A	FAVF 160	Kent	Ahome	MT774581	MT782085	MT782105
*Neopestalotiopsis* sp. Clade B	FAVF 204	Kent	El Rosario	MT774583	MT782087	MT782107
*Neopestalotiopsis* sp. Clade B	FAVF 155	Kent	Ahome	MT774586	MT782090	MT782110
*Neopestalotiopsis* sp. Clade B	FAVF 194	Ataulfo	Ahome	MT774585	MT782089	MT782109
*Neopestalotiopsis* sp. Clade B	FAVF 157	Kent	Ahome	MT774587	MT782091	MT782111
*Neopestalotiopsis* sp. Clade B	FAVF 165	Ataulfo	Ahome	MT774582	MT782086	MT782106
*Neopestalotiopsis* sp. Clade C	FAVF 163	Kent	Ahome	MT774577	MT782081	MT782101
*Neopestalotiopsis* sp. Clade C	FAVF 190	Kent	Ahome	MT774579	MT782083	MT782103
*Neopestalotiopsis* sp. Clade C	FAVF 164	Kent	Ahome	MT774576	MT782080	MT782100
*Neopestalotiopsis* sp. Clade C	FAVF 172	Ataulfo	Ahome	MT774580	MT782084	MT782104
*Neopestalotiopsis* sp. Clade C	FAVF 162	Ataulfo	Ahome	MT774574	MT782078	MT782098
*Neopestalotiopsis* sp. Clade C	FAVF 158	Kent	Ahome	MT774575	MT782079	MT782099
*Neopestalotiopsis* sp. Clade C	FAVF 159	Kent	Ahome	MT774578	MT782082	MT782102
*Neopestalotiopsis* sp. Clade D	FAVF 173	Tommy Atkins	Ahome	MT774572	MT782076	MT782096
*Neopestalotiopsis* sp. Clade D	FAVF 187	Kent	Ahome	MT774568	MT782072	MT782092
*Neopestalotiopsis* sp. Clade D	FAVF 176	Kent	Ahome	MT774569	MT782073	MT782093
*Neopestalotiopsis* sp. Clade D	FAVF 174	Kent	Ahome	MT774570	MT782074	MT782094
*Neopestalotiopsis* sp. Clade D	FAVF 169	Kent	Ahome	MT774571	MT782075	MT782095
*Neopestalotiopsis* sp. Clade D	FAVF 167	Kent	Ahome	MT774573	MT782077	MT782097

**Table 2:** Morphological and cultural characteristics of 20 *Neopestalotiopsis* isolates obtained from mango leaves in this study.

			Average Size (µm) of Conidia and Appendages					
Species ^w^	Origin	Colony Type ^x^	Length	Width	Length of Apical Appendages	Length of Basal Appendage	Number of Apical Appendages	Average Growth (mm day^−1^) ^y^
*Neopestalotiopsis* sp. Clade A (FAVF 160 and FAVF 198)	Ahome, El Rosario	1, 2	21.78 ± 1.61 A	7.23 ± 0.55 A	17.81 ± 2.54 A	5.81 ± 1.31 B	2–3	2.77 ± 0.06 B
*Neopestalotiopsis* sp. Clade B (FAVF 155, FAVF 157, FAVF 165, FAVF 194, and FAVF 204)	Ahome, El Rosario	1, 4	20.93 ± 1.86 A	7.71 ± 0.80 B	18.95 ± 3.68 A	6.89 ± 1.66 C	2–3	2.12 ± 0.16 A
*Neopestalotiopsis* sp. Clade C (FAVF 158, FAVF 159, FAVF 162, FAVF 163, FAVF 164, FAVF 172 and FAVF 190)	Ahome	1, 3	21.20 ± 1.92 A	7.81 ± 0.99 B	21.21 ± 4.71 B	4.77 ± 1.36 A	2–4	2.40 ± 0.23 AB
*Neopestalotiopsis* sp. Clade D (FAVF 167, FAVF 169, FAVF 173, FAVF 174, FAVF 176 and FAVF 187)	Ahome	1, 2, 4	23.29 ± 2.09 B	7.13 ± 0.61 A	18.35 ± 3.35 A	5.24 ± 1.06 B	2–4	2.48 ± 0.19 AB

^w^ Codes within parentheses list the strains isolated for each clade. ^x^ Four colony morphotypes, where colony; 1 = bright-white colony, cottony texture, intermediate mycelium density, acervuli in the middle, regular edges; 2 = bone-white colony, velvety texture, high mycelium density, no acervuli, regular edges; 3 = white colony, powdery spiky texture, intermediate mycelium density, acervuli in the middle, irregular edges; 4 = white colony, powdery texture, low mycelium density, scattered acervuli, regular edges. ^y^ Rate of growth on potato dextrose agar at 25 °C in darkness. Data of averages represent means ± SD. Same letters in the column mean that there are no significant differences between clades, according to LSD in the Tukey test at the *p* ≤ 0.05 level.

## References

[1] Aguirre-Güitrón L., Calderón-Santoyo M., Bautista-Rosales P.U., Ragazzo-Sánchez J.A. (2019). Application of powder formulation of *Meyerozyma caribbica* for postharvest control of *Colletotrichum gloeosporioides* in mango (*Mangifera indica* L.). J. Food Sci. Technol..

[2] Zahedi S.M., Hosseini M.S., Karimi M., Ebrahimzadeh A. (2019). Effects of postharvest polyamine application and edible coating on maintaining quality of mango (*Mangifera indica* L.) cv. Langra during cold storage. Food Sci. Nutr..

[3] Gu C., Yang M., Zhou Z., Khan A., Cao J., Cheng G. (2019). Purification and characterization of four benzophenone derivatives from *Mangifera indica* L. leaves and their antioxidant, immunosuppressive and α-glucosidase inhibitory activities. J. Funct. Foods.

[4] Ibarra-Garza I.P., Ramos-Parra A.P., Hernández-Brenes C., Jacobo-Velázquez D.A. (2015). Effects of postharvest ripening on the nutraceutical and physicochemical properties of mango (*Mangifera indica* L. cv Keitt). Postharvest Biol. Tech..

[5] Ekanayake G., Abeywickrama K., Daranagama A., Kannangara S. (2019). Morphological characterization and molecular identification of stem-end rot associated fungal species isolated from ‘*Karutha Colomban*’ mango fruits in Sri Lanka. J. Agric. Sci-Sri Lanka.

[6] FAO Food and Agriculture Organizations of the United Nations. http://www.fao.org/3/ca5692en/CA5692EN.pdf.

[7] SIAP Servicio de Información Agroalimentaria y Pesquera. Producción Agrícola. https://www.gob.mx/siap/acciones-y-programas/produccion-agricola-33119.

[8] Ploetz R., Freeman S., Litz R. (2009). Foliar, floral and soilborne diseases. The Mango: Botany, Production and Uses.

[9] Otero-Colina G., Rodríguez-Alvarado G., Fernández-Pavía S., Maymon S.M., Ploetz R.C., Aoki T. (2010). Identification and characterization of a novel etiological agent of mango malformation disease in Mexico, *Fusarium mexicanum* sp. nov. Phytopathology.

[10] Félix-Gastélum R., Herrera-Rodríguez G., Martínez-Valenzuela C., Longoria-Espinoza R.M., Maldonado-Mendoza I.E., Quiroz-Figueroa F.R., Espinosa-Matías S. (2013). First report of powdery mildew (*Pseudoidium anacardii*) of mango trees in Sinaloa, Mexico. Plant Dis..

[11] Sandoval-Sánchez M., Nieto-Ángel D., Sandoval-Islas J., Téliz-Ortiz D., Orozco-Santos M., Silva-Rojas H.V. (2013). Fungi associated to stem-end rot and dieback of mango (*Mangifera indica* L.). Agrociencia.

[12] Noriega-Cantú D., Téliz-Ortiz D., Mora-Aguilera A., Mora-Aguilera G., Mora-Aguilera G., Noriega-Cantú D., Pérez-Barraza M. (2017). Enfermedades del mango. El Mango: Su Cultivo, Fitosanidad y Comercialización.

[13] Tovar-Pedraza J.M., Mora-Aguilera J.A., Nava-Díaz C., Lima N.B., Michereff S.J., Sandoval-Islas J.S., Leyva-Mir S.G. (2020). Distribution and pathogenicity of *Colletotrichum* species associated with mango anthracnose in Mexico. Plant Dis..

[14] Mordue J.E.M. (1980). Pestalotiopsis Mangiferae.

[15] Ko Y., Yao K., Chen C., Lin C. (2007). First report of gray leaf spot of mango (*Mangifera indica*) caused by *Pestalotiopsis mangiferae* in Taiwan. Plant Dis..

[16] Ismail A., Cirvilleri G., Polizzi G. (2013). Characterization and pathogenicity of *Pestalotiopsis uvicola* and *Pestalotiopsis clavispora* causing grey leaf spot of mango (*Mangifera indica* L.) in Italy. Eur. J. Plant Pathol..

[17] Maharachchikumbura S.S.N., Hyde K.D., Groenewald J.Z., Xu J., Crous P. (2014). *Pestalotiopsis* revisited. Stud. Mycol..

[18] Maharachchikumbura S.S.N., Laringnonl P., Hyde K.D., Al-Sady A., Liu Z. (2016). Characterization of *Neopestalotiopsis*, *Pestalotiopsis* and *Truncatella* species associated with grapevine trunk diseases in France. Phytopathol. Mediterr..

[19] Hyde K.D., Nilsson R.H., Alias S.A., Ariyawansa H.A., Blair J.E., Cai L., Gorczak M. (2014). One stop shop: Backbones trees for important phytopathogenic genera: I (2014). Fungal Divers..

[20] Jayawardena R.S., Hyde K.D., McKenzie E.H., Jeewon R., Phillips A.J.L., Perera R.H., Tennakoon D.S. (2019). One stop shop III: Taxonomic update with molecular phylogeny for important phytopathogenic genera: 51–75 (2019). Fungal Divers..

[21] Jayawardena R.S., Zhang W., Liu M., Maharachchikumbura S.S.N., Zhou Y., Huang J., Hyde K.D. (2015). Identification and characterization of *Pestalotiopsis*-like fungi related to grapevine diseases in China. Fungal Biol..

[22] Ayoubi N., Soleimani M.J. (2016). Morphological and molecular identification of *Neopestalotiopsis asiatica* causing leaf spot on sweet almond. J. Plant Pathol..

[23] Chamorro M., Aguado A., De los Santos B. (2016). First report of root and crown rot caused by *Pestalotiopsis clavispora* (*Neopestalotiopsis clavispora*) on strawberry in Spain. Plant Dis..

[24] Li L., Pan H., Chen M., Zhong C. (2016). First report of *Pestalotiopsis microspora* causing postharvest rot of kiwifruit in Hubei province, China. Plant Dis..

[25] Akinsanmi O., Nisa S., Jeff-Ego O., Shivas R., Drenth A. (2017). Dry flower disease of Macadamia in Australia caused by *Neopestalotiopsis macadamiae* sp. nov. and *Pestalotiopsis macadamiae* sp. nov. Plant Dis..

[26] Solarte F., Muñoz C.G., Maharachchikumbura S.S.N., Álvarez E. (2018). Diversity of *Neopestalotiopsis* and *Pestalotiopsis* spp., causal agents of guava scab in Colombia. Plant Dis..

[27] Tsai I., Maharachchikumbura S.S.N., Hyde K.D., Ariyawansa H.A. (2018). Molecular phylogeny, morphology and pathogenicity of *Pseudopestalotiopsis* species on *Ixora* in Taiwan. Mycol. Prog..

[28] Okigbo R., Osuinde M. (2003). Fungal leaf spot diseases of mango (*Mangifera indica* L.) in southeastern Nigeria and biological control with *Bacillus subtilis*. Plant Protect. Sci..

[29] Maharachchikumbura S.S.N., Zhang Y., Wang Y., Hyde K.D. (2013). *Pestalotiopsis anacardiacearum* sp. 29. nov. (*Amphisphaeriaceae*) has an intricate relationship with *Penicillaria jocosatrix*, the mango tip borer. Phytotaxa.

[30] Crous P.W., Wingfield M.J., Le Roux J.J., Richardson D.M., Strasberg D., Shivas R.G., Sonawane M.S. (2015). Fungal Planet description sheets: 371–399. Persoonia.

[31] Maharachchikumbura S.S.N., Guo L.D., Cai L., Chukeatirote E., Wu W.P., Sun X., Hyde K.D. (2012). A multi-locus backbone tree for *Pestalotiopsis*, with a polyphasic characterization of 14 new species. Fungal Divers..

[32] Liu F., Hou L., Raza M., Cai L. (2017). *Pestalotiopsis* and allied genera from *Camellia*, with description of 11 new species from China. Sci. Rep..

[33] Nozawa S., Seto Y., Watanabe K. (2019). First report of leaf blight caused by *Pestalotiopsis chamaeropis* and *Neopestalotiopsis* sp. in Japanese andromeda. J. Gen. Plant. Pathol..

[34] Wang Y., Xiong F., Lu Q., Hao H., Zheng M., Wang L., Yang Y. (2019). Diversity of *Pestalotiopsis*-like species causing gray blight disease of tea plants (*Camellia sinensis*) in China, including two novel *Pestalotiopsis* species, and analysis of their pathogenicity. Plant Dis..

[35] Belisário R., Aucique-Pérez C.E., Abreu L.M., Salcedo S.S., Oliveira W.M., Furtado G.Q. (2020). Infection by *Neopestalotiopsis* spp. occurs on unwounded eucalyptus leaves and is favoured by long periods of leaf wetness. Plant Pathol..

[36] Cai L., Giraud T., Zhang N., Begerow D., Cai G., Shivas R.G. (2011). The evolution of species concepts and species recognition criteria in plant pathogenic fungi. Fungal Divers..

[37] Shu J., Yu Z., Sun W., Zhao J., Li Q., Tang L., Luo S. (2020). Identification and characterization of pestalotioid fungi causing leaf spots on mango in southern China. Plant Dis..

[38] Patil V.A., Mehta B.P., Deshmukh A.J., Bavalgave V.G. (2019). Fungicides for the management of gray leaf blight (*Pestalotia anacardii*) of mango. Int. J. Econ. Plants..

[39] Thaochan N., Pornsuriya C., Chairin T., Sunpapao A. (2020). Roles of systemic fungicide in antifungal activity and induced defence responses in rubber tree (*Hevea brasiliensis*) against leaf fall disease caused by *Neopestalotiopsis cubana*. Physiol. Mol. Plant P..

[40] Tippeshi L.C., Suryanarayana V., Naik S.T. (2010). Survey and management of *Pestalotiopsis* leaf blight of *Jatropha* – a destructive new disease in Karnataka. Indian Phytopath..

[41] Mahadevakumar S., Janardhana G.R. (2014). First report of *Pestalotiopsis* species causing leaf spot of cowpea (*Vigna unguiculata*) in India. Plant Dis..

[42] Mahadevakumar S., Janardhana G.R. (2014). First report on the association of *Pestalotiopsis mangiferae* with leaf blight disease of *Canthium dicoccum* in India. For. Pathol..

[43] Niu X.Q., Zhu H., Yu F.Y., Tang Q.H., Song W.W., Liu L., Qin W.Q. (2015). First report of *Pestalotiopsis menezesiana* causing leaf blight of coconut in Hainan, China. Plant Dis..

[44] Chen Y., Zhang A.F., Yang X., Gu C.Y., Kyaw E.P. (2016). First report of *Pestalotiopsis clavispora* causing twig blight on highbush blueberry (*Vaccinium corymbosum*) in Anhui province of China. Plant Dis..

[45] Madden L., Hughes G., van den Bosch F. (2007). The Study of Plant Disease Epidemics.

[46] Freeman S., Katan T., Shabi E. (1998). Characterization of *Colletotrichum* species responsible for anthracnose disease of various fruits. Plant Dis..

[47] Zhang Y., Maharachchikumbura S.S.N., McKenzie E.H.C., Hyde K.D. (2012). A novel species of *Pestalotiopsis* causing leaf spots of *Trachycarpus fortunei*. Cryptogamie Mycol..

[48] Zervakis G., Philippoussis A., Ioannidou S., Diamantopoulou P. (2001). Mycelium growth kinetics and optimal temperature conditions for the cultivation of edible mushroom species on lignocellulosic substrates. Folia Microbiol..

[49] Di Rienzo J.A., Casanoves F., Balzarini G., González L., Tablada M., Robledo W. (2014). InfoStat, Versión 2014.

[50] White T., Bruns T., Lee S., Taylor J., Innis M., Gelfand D., Sninsky J., White T. (1990). Amplification and direct sequencing of fungal ribosomal RNA genes for phylogenetics. PCR Protocols: A Guide to Methods and Applications.

[51] Carbone I., Kohn L.M. (1999). A method for designing primer sets for speciation studies in filamentous ascomycetes. Mycologia.

[52] Glass N.L., Donaldson G.C. (1995). Development of primer sets designed for use with the PCR to amplify conserved genes from filamentous ascomycetes. Appl. Environ. Microbiol..

[53] O’Donnell K., Cigelnik E. (1997). Two divergent intragenomic rDNA ITS2 types within a monophyletic lineage of the fungus *Fusarium* are nonorthologous. Mol. Phylogenet. Evol..

[54] Kumar S., Stecher G., Tamura K. (2016). MEGA7: Molecular evolutionary genetics analysis version 7.0 for bigger datasets. Mol. Biol. Evol..

[55] Silvestro D., Michalak I. (2012). raxmlGUI: A graphical front-end for RAxML. Org. Divers. Evol..

[56] Sauceda-Acosta C.P., Lugo-García G.A., Villaseñor-Mir H.E., Partida-Ruvalcaba L., Reyes-Olivas A. (2015). Un método preciso para medir severidad de roya de la hoja (*Puccinia triticina* Eriksson) en trigo. Rev. Fitotec. Mex..

